# API5-Hsp20 axis regulate apoptosis and viral infection in mud crab (*Scylla paramamosain*)

**DOI:** 10.3389/fmicb.2023.1323382

**Published:** 2023-12-08

**Authors:** Hang Hu, Nan Deng, Xinshan Zhao, Cheng Yi, Weiqian Wei, Yi Gong

**Affiliations:** ^1^School of Life Sciences, Nanchang University, Nanchang, China; ^2^Jiangxi Provincial Key Laboratory of Aquatic Resources and Utilization, Nanchang University, Nanchang, China; ^3^Guangdong Provincial Key Laboratory of Marine Biology, Shantou University, Shantou, China

**Keywords:** *Scylla paramamosain*, WSSV, API5, apoptosis, innate immunity

## Abstract

Apoptosis Inhibitor 5 (API5) is a widely concerned nuclear protein with diverse functions in organisms, so far, study of API5 is still quite limited in lower animals, and its role in viral immune response has not been addressed. Here, we explored the function of API5 in mud crab (*Scylla paramamosain*) during White Spot Syndrome Virus (WSSV) infection. The interacting protein Hsp20 of API5 was screened by pull-down assay, and API5 and hsp20 were knocked down by RNAi interference. The results showed that API5 was upregulated along with virus infection, silencing of API5 led to increased WSSV copy numbers and apoptotic rate of hemocytes, highlighting its significance in the immune response. Moreover, we discovered a novel interaction between API5 and Heat Shock Protein 20 (Hsp20), and then revealed that Hsp20 could promote cell apoptosis of hemocytes and reduce viral copy numbers by suppressing API5. The current study therefore improves the knowledge of API5-Hsp20 axis and provides novel insights into intricate mechanisms governing the antiviral response in marine crustaceans.

## Introduction

Numerous studies in the past have indicated the presence of various forms of immune memory in invertebrates (Netea et al., 2011; Criscitiello and Figueiredo, 2013; Netea and Meer, 2017). Since invertebrates lack the typical adaptive immune system, characterized by the absence of lymphocytes and antibodies, this form of immune memory has been termed innate immune memory (Kurtz, 2005) or immune priming (Little and Kraaijeveld, 2004). Apoptosis, regarded as a cellular immune response, entails programmed cell death featuring phenomena such as plasma membrane blebbing, cytoplasmic shrinkage, and chromatin condensation (Clarke and Tyler, 2009). In both vertebrates and invertebrates, viral infections can lead to apoptosis in infected host cells (Campisi et al., 2016). Viral infection can induce cell death in infected cells by activating the apoptosis pathway, thereby inhibiting viral replication and enhancing the host immune response (Orzalli and Kagan, 2017). To date, research on apoptosis and viral infection has predominantly focused on higher animals, with limited studies, especially in marine invertebrates.

Apoptosis inhibitor 5 (API5, also known as AAC-11 or FIF) is a nuclear protein initially identified for its role in preventing cell apoptosis following growth factor deprivation (Imre and Rajalingam, 2018). Structural analysis reveals that API5, as a protein–protein interaction mediator, consists of multiple helices and comprises HEAT and ARM repeat protein-binding modules (Han et al., 2012). In existing studies, API5 has been found consistently upregulated in various cancer cells, including non-small cell lung cancer (Sasaki et al., 2001), breast cancer (Kuttanamkuzhi et al., 2023), cervical cancer (Kim et al., 2000), and B-cell chronic lymphocytic leukemia (Krejci et al., 2007). Research suggests that API5 serves as a direct inhibitor of caspase-2 by binding to the CARD domain of Caspase-2, thereby impeding its dimerization and activation, affecting cell apoptosis (Imre et al., 2017). Other studies indicate that API5 inhibits BIM protein degradation through the FGF2-FGFR1-PKCδ-Erk signaling pathway or upregulates FGF2 and activates downstream pathways involving FGFR1, PKCδ, and ERK, ultimately leading to ubiquitin-dependent degradation of BIM, thereby attenuating apoptosis in tumor cells (Noh et al., 2014). The increased expression of API5 in tumor cells may contribute to resistance to cisplatin drug therapy (Jang et al., 2017). Recent research suggests that API5 protein in the human intestine enhances the survival of Paneth cells, potentially offering new therapeutic avenues for treating Crohn disease (Matsuzawa-Ishimoto et al., 2022). So far, research focused on API5 is still quite limited in lower animals, and its role in viral immune response has not been intensively explored.

Hsp20 belongs to the small heat shock protein (sHsp) family, comprising 10 different sHsps with molecular weights ranging from 12 to 43 kDa (Kappé et al., 2003). This protein is detectable in all tissues but is most abundant in the heart, smooth muscle, and skeletal muscle (Fan et al., 2005). Recent research has highlighted Hsp20 as a hot topic in cancer research due to its regulation of proliferation and apoptosis (Li et al., 2019). For instance, in breast cancer, Hsp20 suppresses BC cell proliferation and promotes BC cell apoptosis by inhibiting the MAPK and AKT signaling pathways (Yang et al., 2022). In liver cancer, direct interaction between Hsp20 and Bax protein promotes HCC cell apoptosis (Nagasawa et al., 2014). Moreover, Hsp20 plays a crucial role in cardiac protection, with Hsp20 phosphorylation having a protective effect in myocardial ischemia/reperfusion injury (Qian et al., 2009) and in cardiac fibrosis (Gardner et al., 2019). Hsp20 has also been shown to be regulated by microRNA-320, influencing cardiac pathophysiology (Ren et al., 2009).

In an attempt to explore the role of API5 in apoptosis regulation and antiviral immune response in marine invertebrates, *Sp*API5 identified in mud crab (*Scylla paramamosain*) was characterized in this study. The results showed that API5 could regulate apoptosis and virus infection by cooperating with Hsp20, which improves our knowledge of API5-Hsp20 axis in the antiviral innate immune response of marine crustaceans.

## Results

### Amino acid sequence bioinformatics of *Sp*API5

**Figure 1:**
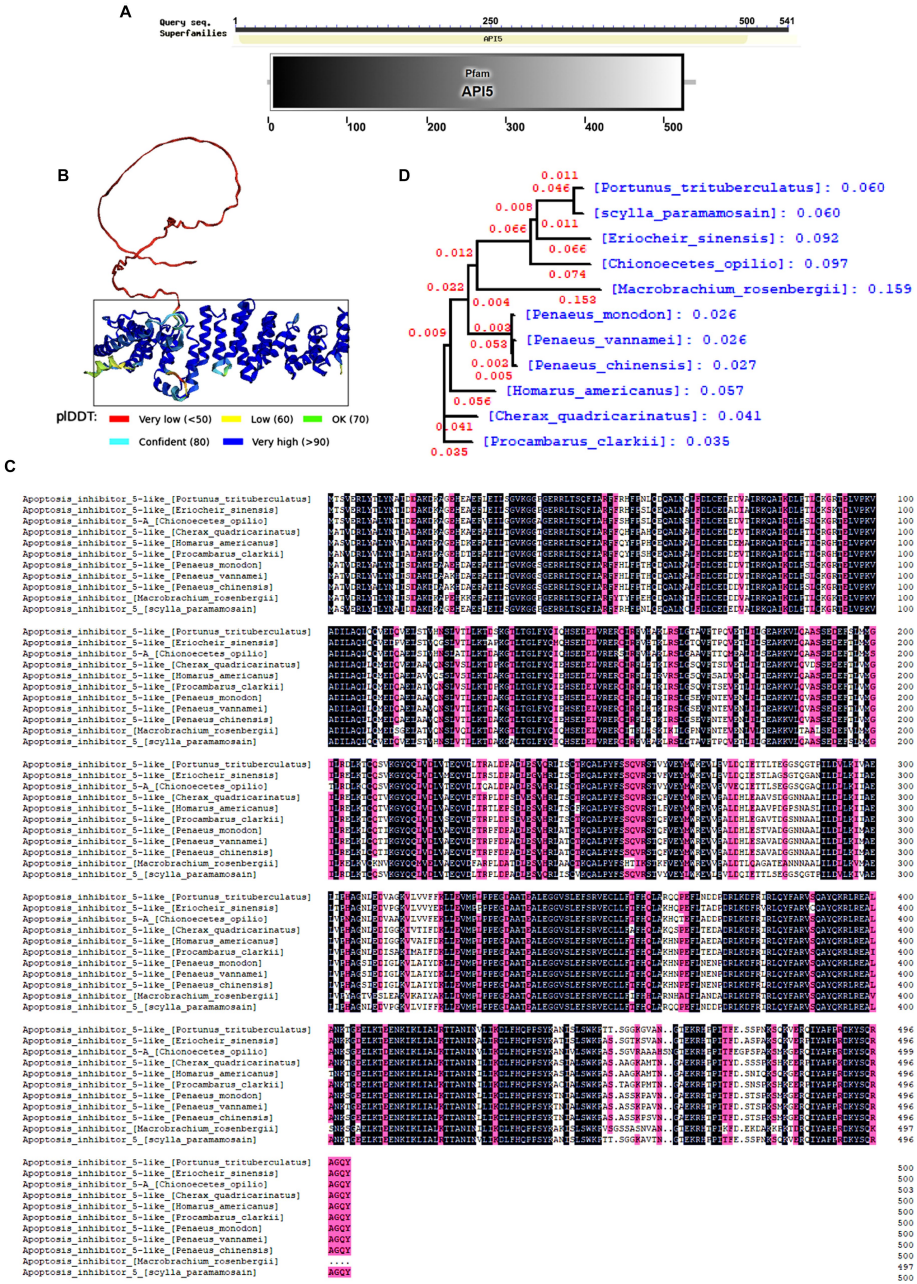
Bioinformatics analysis of *Sp*API5. **(A)** *Sp*API5 domains predicted by Smart online tool. **(B)** Tertiary structure of *Sp*API5 predicted by Alpha fold online tool, API5 domain is marked with a black rectangle. **(C)** Multiple alignments of *Sp*API5. *Pt*: *Portunus trituberculatus* (MPC22527.1), *Pv: Pen aeus vannamei* (XP_027206983.1), *Tl*: *Trinorchestia longiramus* (KAF2356821.1), *Ha*: *Hyalella azaateca* (XP_018022038.1), *Hk*: *Hyposmocoma kahamanoa* (XP_02631 8966.1), *Vt*: *Vanessa tameamea* (XP_026498634.1), *Sl*: *Spodoptera litura* (XP_02283 5907.1), *Ls*: *Leptidea sinapis* (VVC87739.1), *Ha*: *Helicoverpa armigera* (XP_021181 674.1), *Ah*: *Aphantopus hyperantus* (XP_034829298.1); **(D)** Phylogenetic analysis (neighbor joining analysis) of *Sp*API5.

The open reading frame (ORF) of *Sp*API5 comprises 1,625 bp of nucleic acid, which translates into a sequence of 542 amino acids (Gene Bank accession number. OR670539). The protein has an estimated molecular mass of approximately 60.65 kDa. The API5 family domain within the amino acid sequence was predicted using the Smart online tool (Figure 1A). Besides, the tertiary structure of the protein was predicted by the alpha fold2 online tool (Figure 1B). Moreover, DNAMAN software was employed for conducting multi-species sequence comparison and evolutionary tree analysis. The results revealed a relatively conserved API5 gene across various invertebrate species (Figure 1C), the phylogenetic tree also indicated that both the *S. paramamosain* and *P. trisulatus* belong to the same clade (Figure 1D).

### Expression of API5 protein and preparation of polyclonal antibody

**Figure 2:**
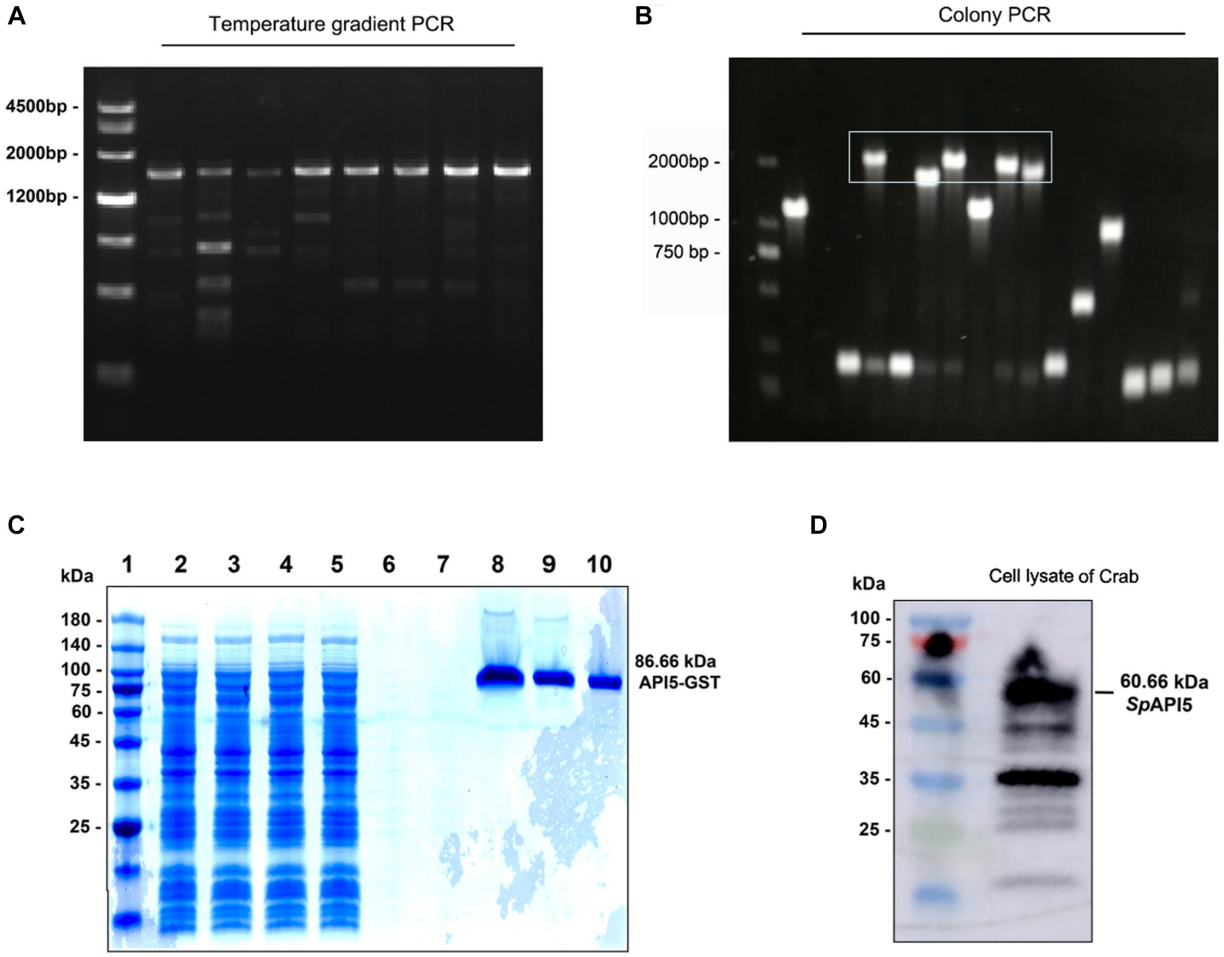
Prokaryotic expression of API5 protein and preparation of API5 antibody. **(A)** Utilizing temperature gradient (50°C–60°C) PCR for identification of the optimal amplification temperature. **(B)** After transfection of the recombinant plasmid into BL21, positive colonies were selected using PCR screening. **(C)** Coomassie brilliant blue staining was employed to assess the level of *Sp*API5 protein induction and purification status. Lane 1: 8–180 kDa marker; Lane 2–5: Sample effluent; Lane 6: Wash effluent; Lane 7–10: Elution effluent. **(D)** The specificity of API5 antibodies to hemocytes was assessed using Western blot analysis.

Through temperature gradient (50°C–60°C) PCR, an appropriate temperature 60°C for PCR was selected, and a 1,625 bp band of API5 gene was determined (Figure 2A). Then the positive clones were screened by colony PCR (Figure 2B). The molecular weight of the GST-tagged API5 recombinant protein was determined to be 86.66 kDa (Figure 2C). After that, western blot analysis was employed to detect the specificity of polyclonal antibodies obtained by injecting mice with purified recombinant protein, the results indicated that the band corresponding to the anti-API5 protein had a molecular weight of 60.66 kDa, and it exhibited superior specificity compared to other bands (Figure 2D). Therefore, it is evident that this antibody possesses high specificity and can be utilized in subsequent research to assess the protein levels of API5.

### Expression of API5 in mud crab during WSSV infection

**Figure 3:**
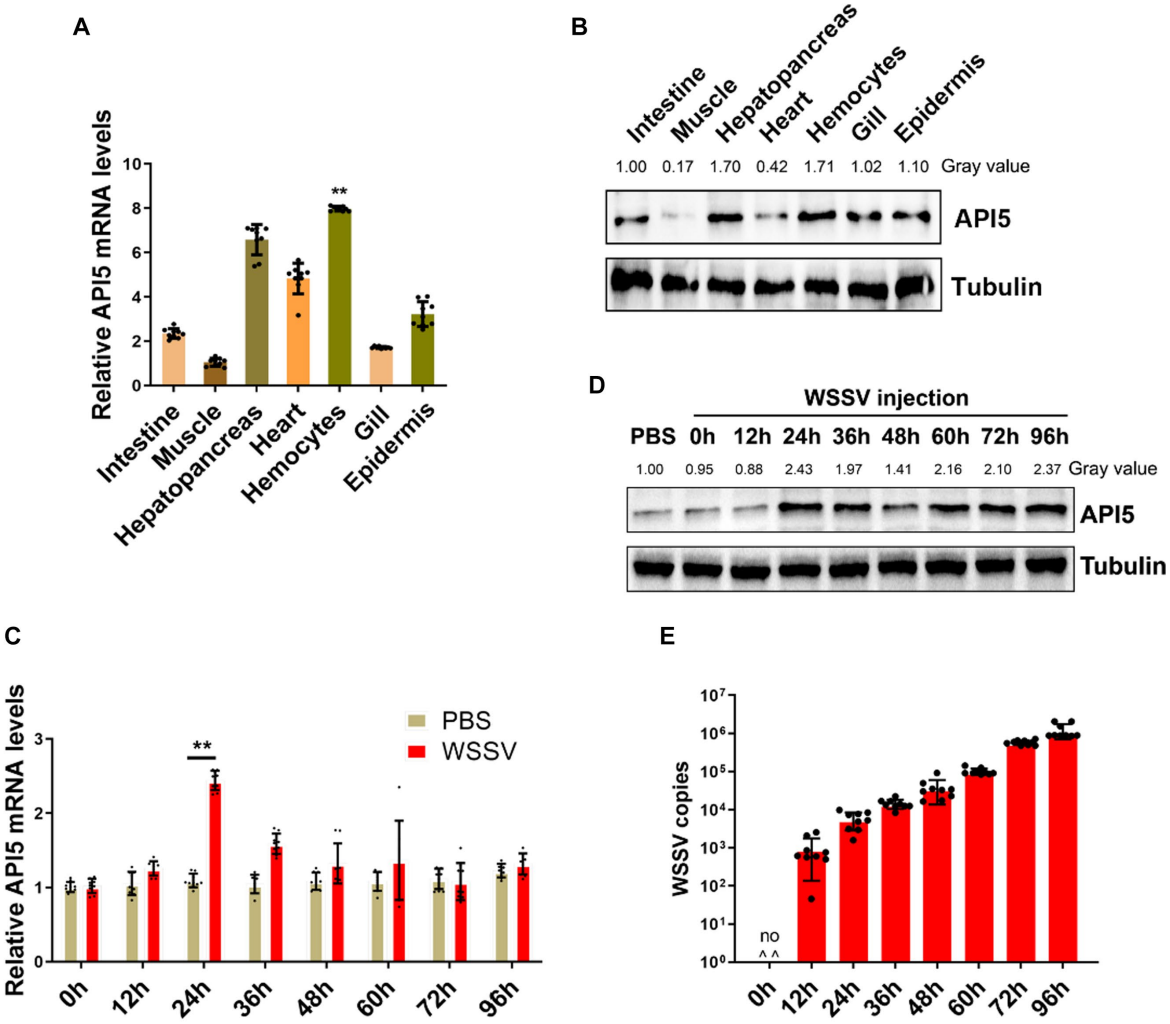
The response of API5 in mud crab subjected to WSSV infection. **(A,B)** The distribution of API5 mRNA and protein in various tissues of mud crabs, including intestine, epidermis, heart, hepatopancreas, muscle, gill and hemocytes, which was assessed by qRT-PCR **(A)** and western blot **(B)**, respectively. **(C,D)** The mRNA and protein expressions of API5 in mud crab during WSSV infection. Mud crabs were treated with WSSV for different time, then hemocytes was collected and measured using qRT-PCR **(C)** and western blot **(D)**, respectively. PBS was used as the control group. **(E)** WSSV copy numbers in mud crabs during virus infection at different time point. Mud crabs were injected with WSSV for 12, 24, 36, 48, 60, 72 and 96 h, then the muscles of mud crabs were collected and subjected to virus particles copy number analysis. The results were based on three parallel experiments, and the data is presented as mean ± SD, all the data were analyzed statistically by student’s *t* test (*p < 0.05 and **p < 0.01).

To investigate role of API5 in mud crab, tissue distribution measurement was conducted, including intestine, muscle, heart, hemocytes, epidermis, hepatopancreas, and gill. The results showed that API5 mRNA was notably higher in hepatopancreas, hemocytes and heart than in other tissues (Figure 3A). Additionally, Western blot analysis revealed API5 protein was overexpressed in epidermis, heart, hepatopancreas, gills and hemocytes compared to other tissues (Figure 3B). To establish whether API5 is involved in immune regulation, AIP5 was detected during WSSV infection in mud crab. Results showed a significant increase in API5 mRNA levels in hemocytes, with the most prominent rise occurring at 24 h post-infection (Figure 3C), a similar trend was observed in API5 protein expression levels (Figure 3D), meanwhile, the copy number of virus particles in different time periods indicated that WSSV replicated smoothly in mud crab (Figure 3E), the above data strongly suggest the association of API5 with antiviral immune response in mud crab.

### API5 promotes viral replication by suppressing apoptosis

**Figure 4:**
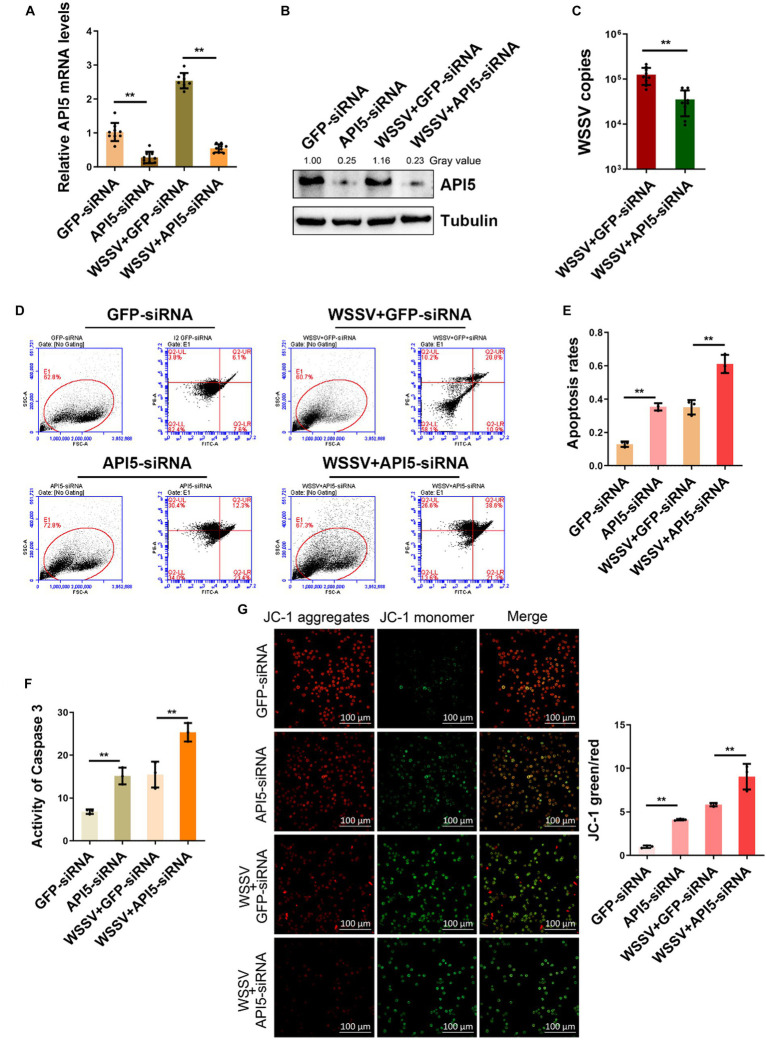
API5 inhibits apoptosis and increase WSSV replication. **(A,B)** Silencing of API5 in mud crab during WSSV infection. WSSV and API5 specific siRNA were co-injected into mud crab for 24 h, then qRT-PCR **(A)** and Western blot **(B)** were performed to assess interference efficiency. **(C)** The effect of API5 silencing on WSSV infection. WSSV and API5 specific siRNA were co-injected into mud crab for 24 h, then WSSV copy numbers of different treatment groups was detected by probe qPCR. **(D–G)** The influence of API5 silencing on apoptosis during WSSV infection. WSSV and API5 specific siRNA were co-injected into mud crab for 24 h, then the apoptotic rate of hemocytes were assessed using flow cytometry with Annexin V staining **(D,E)**, caspase 3 activity detection **(F)** and mitochondrial Δψm assay included confocal microscope observation and microplate reader detection **(G)**, respectively. Data represents mean ± s.d. of triplicate assays, all the data were analyzed statistically by student’s *t* test (*p < 0.05 and **p < 0.01).

To further validate the immunological role of API5 in mud crab, we employed RNAi to silence API5 in mud crab during WSSV infection. Significant inhibition of both API5 mRNA and protein expression levels has been demonstrated through the utilization of qRT-PCR and Western blotting (Figures 4A,B). Concurrently, after co-injection of WSSV and API5-siRNA, the viral copy numbers in mud crabs were significantly increased compared to the control group (Figure 4C). These data indicate the pivotal role of API5 in the antiviral immune response of mud crab against WSSV infection. Furthermore, to explore the involvement of apoptosis during API5-mediated antiviral process, Annexin V assay was used to assess the apoptotic rate of hemocytes. The results revealed that the apoptotic rate of hemocytes were significantly increased when API5 was silenced during WSSV infection (Figures 4D,E). Recent studies have shown that API5 could induce Caspase-3-dependent apoptosis through E2F1 pathway (Giovanni, 2000; Mayank et al., 2015). Moreover, the API5-mediated NF-κB pathway can also activate BCl2-induced apoptosis (George et al., 2002; Ren et al., 2010), while Bcl family is closely related to mitochondrial membrane potential (Chen et al., 2011). Therefore, we examined the changes of Caspase3 and mitochondrial membrane potential after knocking down API5. Additionally, caspase-3 activity and mitochondrial Δψm assay were also conducted and a similar trend was observed (Figures 4F,G). Taken together, it is conceivable that API5 contributes to WSSV infection by inhibiting apoptosis in mud crab.

### Screening and verification of API5-binding proteins

**Figure 5:**
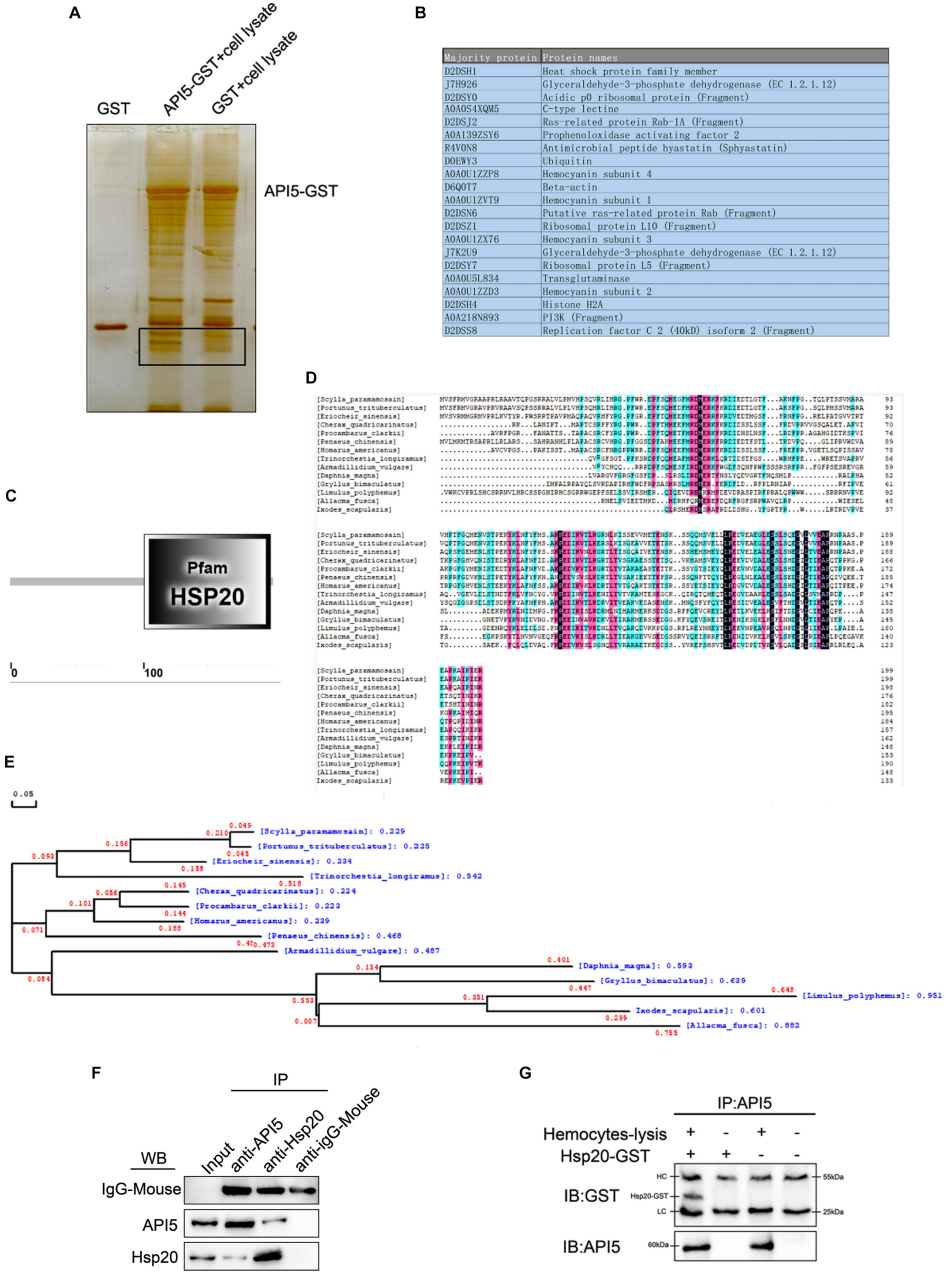
Screening and verification of proteins interacting with *Sp*API5. **(A)** GST pull-down analysis of *Sp*API5, followed by SDS-PAGE silver nitrate staining. **(B)** Mass spectrometry detection of API5 interacting proteins. **(C)** Amino acid sequence and secondary structure prediction of *Sp*Hsp20 open reading frame by Smart online tool. **(D)** Multiple alignments of *Sp*Hsp20. *Pt*: *Portunus trituberculatus* (XP_0451082 41.1), *Es: Eriocheir sinensis* (XP_050685817.1), *Cq*: *Cherax quadricarinatus* (XP_05 3629790.1), *Pc*: *Procambarus clarki* (XP_045592466.1), *Pc*: *Penaeus chinensis* (XP_ 047488002.1), *Ha*: *Homarus america nus* (XP_042221951.1), *Tl*: Trinorchestia longiramus (KAF2362977.1), *Av*: *KAF2362977.1* (RXG54324.1), *Dm*: *Daphnia magna* (KZS15421.1), *Gb*: *Gryllus bimaculatus* (GLH08826.1), *Lp*: *Limulus polyphemus* (XP_013793689.1), *Af: Allacma fuscaIs* (CAG7719130.1), *Is*: *Ixodes scapularis* (AAV63539.1). **(E)** Phylogenetic analysis (neighbor-joining analysis) of *Sp*Hsp20. **(F)** Protein interaction verification between *Sp*API5 and *Sp*Hsp20 through IP analysis, anti-IgG-Mouse was served as the control group. **(G)** API5 as bait protein was pulled down by anti-API5, and the effluent after incubation with the lysate of mud crab hemocytes was detected by GST antibody.

In order to explore the mechanisms of API5-mediated antiviral immune response, API5-binding proteins were screened by GST pull-down experiment, followed by mass spectrometry analysis. A series of proteins with differential expressions were identified (Figures 5A,B). The protein with the highest evaluation score, Hsp20 from the heat shock protein family member, was selected for further analysis. *Sp*Hsp20 is consist of 200 amino acids, and Hsp20 family domain within the amino acid sequence was predicted using the Smart online tool (Figure 5C). Then, DNAMAN software was utilized for multi-species sequence comparison and evolutionary tree analysis. The results revealed that Hsp20 was rarely conserved among invertebrate species (Figure 5D). The phylogenetic tree showed that *Sp*Hsp20 was evolutionarily close to *Portulus trisulatus* (Figure 5E). After that, the interaction between *Sp*API5 and *Sp*Hsp20 was validated through IP (Figure 5F) and co-IP (Figure 5G) assays, the results showed that *Sp*API5 and *Sp*Hsp20 could bind with each other. The above data indicate that API5 can bind with Hsp20 in mud crab.

### Hsp20 negatively regulates API5 during WSSV infection

**Figure 6:**
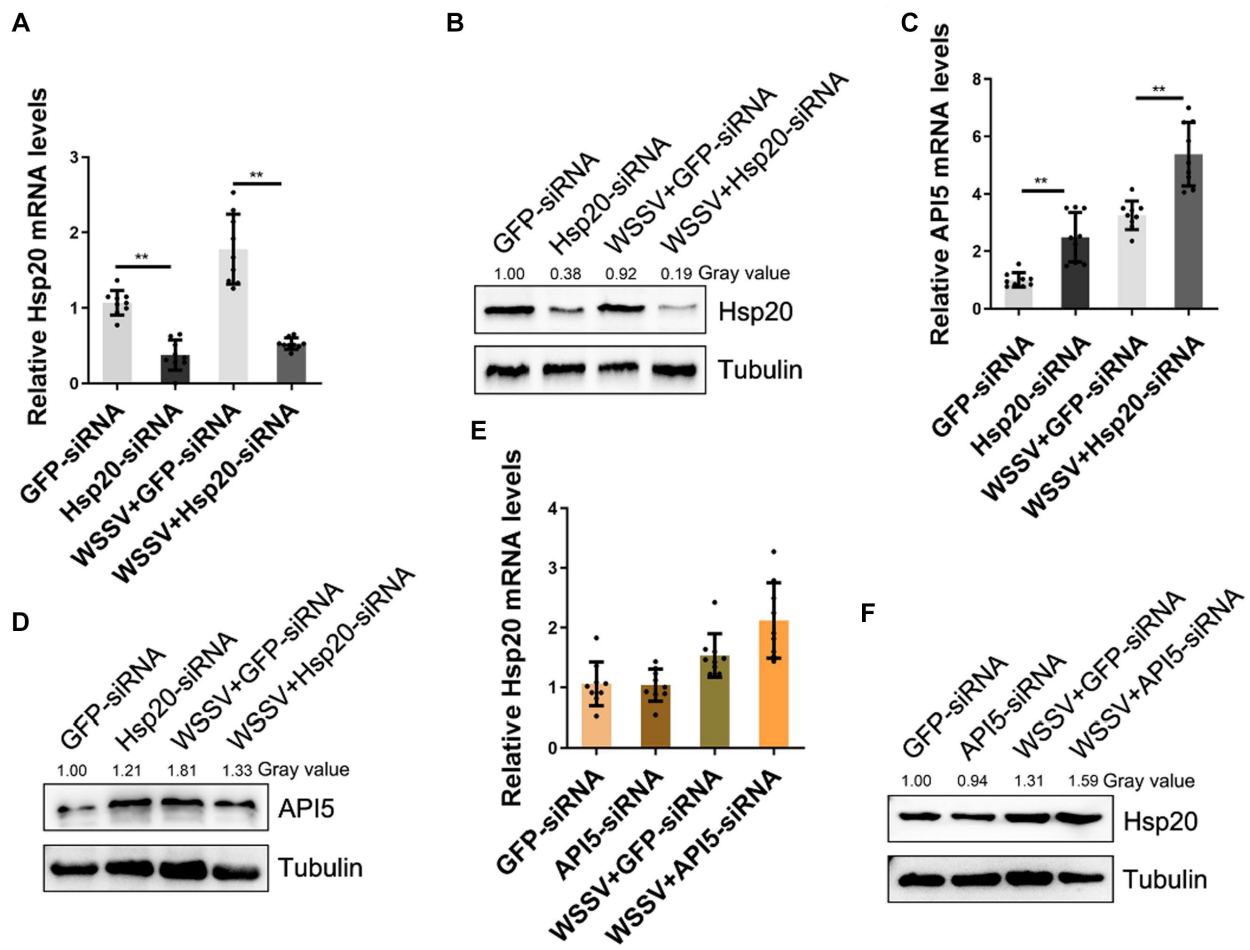
API5 is negatively regulated by Hsp20. **(A,B)** Silencing of Hsp20 in mud crab during WSSV infection. WSSV and Hsp20 specific siRNA were co-injected into mud crab for 24 h, then qRT-PCR **(A)** and Western blot **(B)** were performed to assess interference efficiency. **(C,D)** The influence of Hsp20 silencing on gene expression of API5 during WSSV infection. WSSV and Hsp20-siRNA were co-injected into mud crab, then the mRNA **(C)** and protein **(D)** levels of API5 were examined by qRT-PCR and Western blot, respectively. **(E,F)** The effect of API5 silencing on gene expression of Hsp20 during WSSV infection. WSSV and API5-siRNA were co-injected into mud crab, then the mRNA **(E)** and protein **(F)** levels of Hsp20 were examined by qRT-PCR and Western blot, respectively. The results are based on three parallel experiments and shown as mean values ± SD, all the data were analyzed statistically by student’s *t* test (*p < 0.05 and **p < 0.01).

To further elucidate the relationship between API5 and Hsp20 regulation axis during WSSV infection, RNA interference experiments were conducted. Firstly, the interference efficiency of Hsp20-siRNA was evaluated by qRT-PCR and western-blot respectively, the results showed that both mRNA and protein levels of Hsp20 were significantly suppressed (Figures 6A,B). Then, through silencing API5 and Hsp20, respectively, during WSSV infection, we found that upon WSSV infection and Hsp20-siRNA treatment, API5 exhibited a significant increase in both mRNA expression and protein levels (Figures 6C,D). Conversely, when API5-siRNA was injected in mud crab, Hsp20 mRNA expression level showed no significant difference, and its protein expression remained relatively consistent (Figures 6E,F). Taken together. These data clearly suggest that API5 was negatively regulated by Hsp20 in mud crab.

### Hsp20 suppresses viral replication via promoting apoptosis

**Figure 7:**
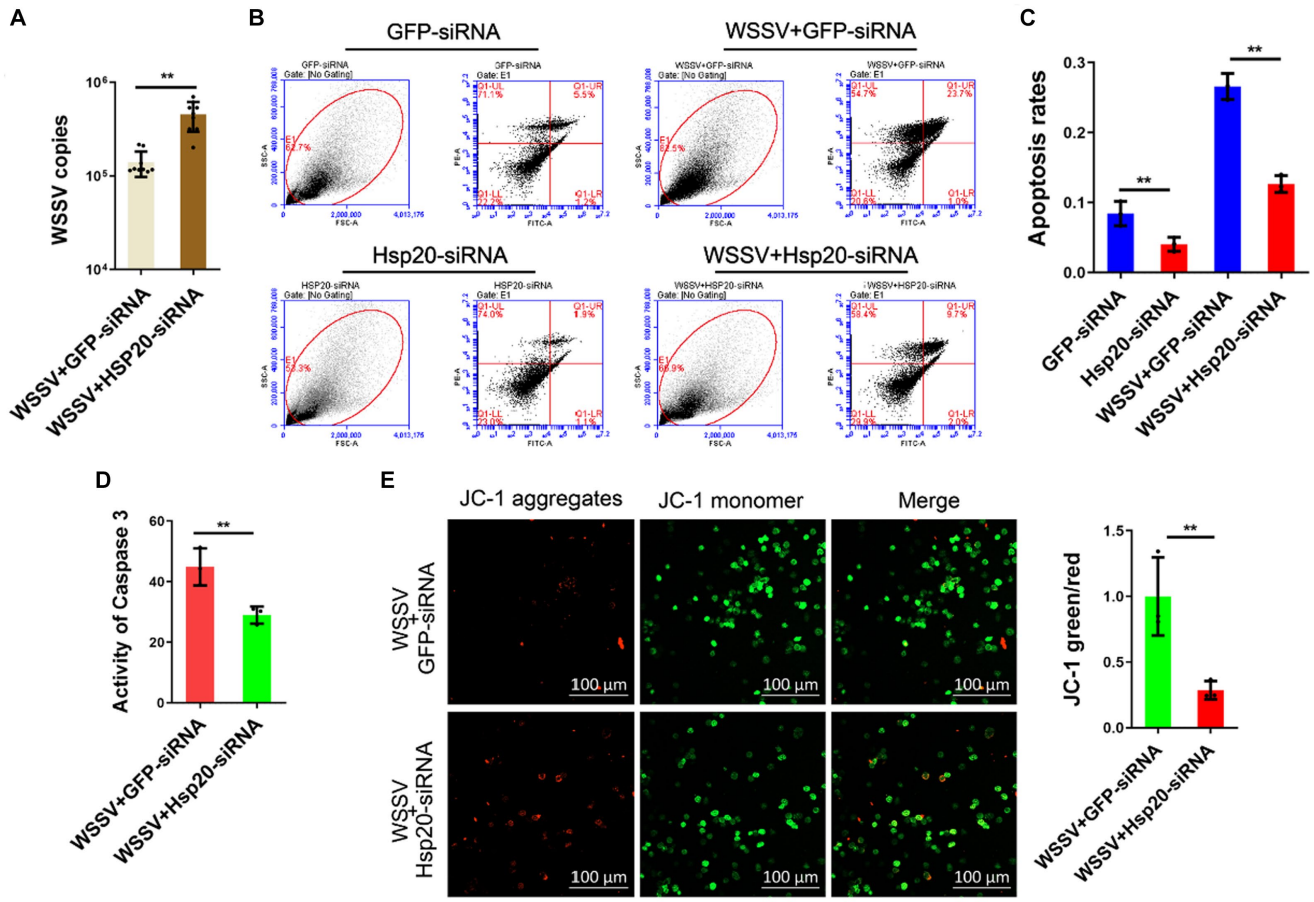
Hsp20 against viral infection by promoting apoptosis. **(A)** The effect of Hsp20 silencing on WSSV infection. WSSV and Hsp20 specific siRNA were co-injected into mud crab for 24 h, then WSSV copy numbers of different treatment groups was detected by probe qPCR. **(B–E)** The influence of Hsp20 silencing on apoptosis during WSSV infection. WSSV and Hsp20 specific siRNA were co-injected into mud crab for 24 h, then the apoptotic rate of hemocytes were assessed using flow cytometry with Annexin V staining **(B,C)**, caspase 3 activity detection **(D)** and mitochondrial Δψm assay included confocal microscope observation and microplate reader detection **(E)**, respectively. The results are based on three parallel experiments and shown as mean values ± SD, all the data were analyzed statistically by student’s *t* test (*p < 0.05 and **p < 0.01).

In order to confirm whether Hsp20 was relevant to API5-mediated antiviral immune response, we knocked down Hsp20 and then detected the influence on virus replication. The results showed that silencing of Hsp20 significantly promote WSSV infection in mud crab (Figure 7A), indicating the positive role of Hsp20 during viral invasion. Then, to explore the involvement of apoptosis in Hsp20-mediated antiviral process, Annexin V assay was used to assess the apoptotic rate of hemocytes. The results revealed that the apoptotic rate of hemocytes were significantly decreased when Hsp20 was silenced during WSSV infection (Figures 7B,C). Additionally, caspase-3 activity and mitochondrial Δψm assay were also conducted and a similar trend was observed (Figures 7D,E). Taken together, it is conceivable that Hsp20 could suppress WSSV infection by promoting apoptosis in mud crab.

**Figure 8:**
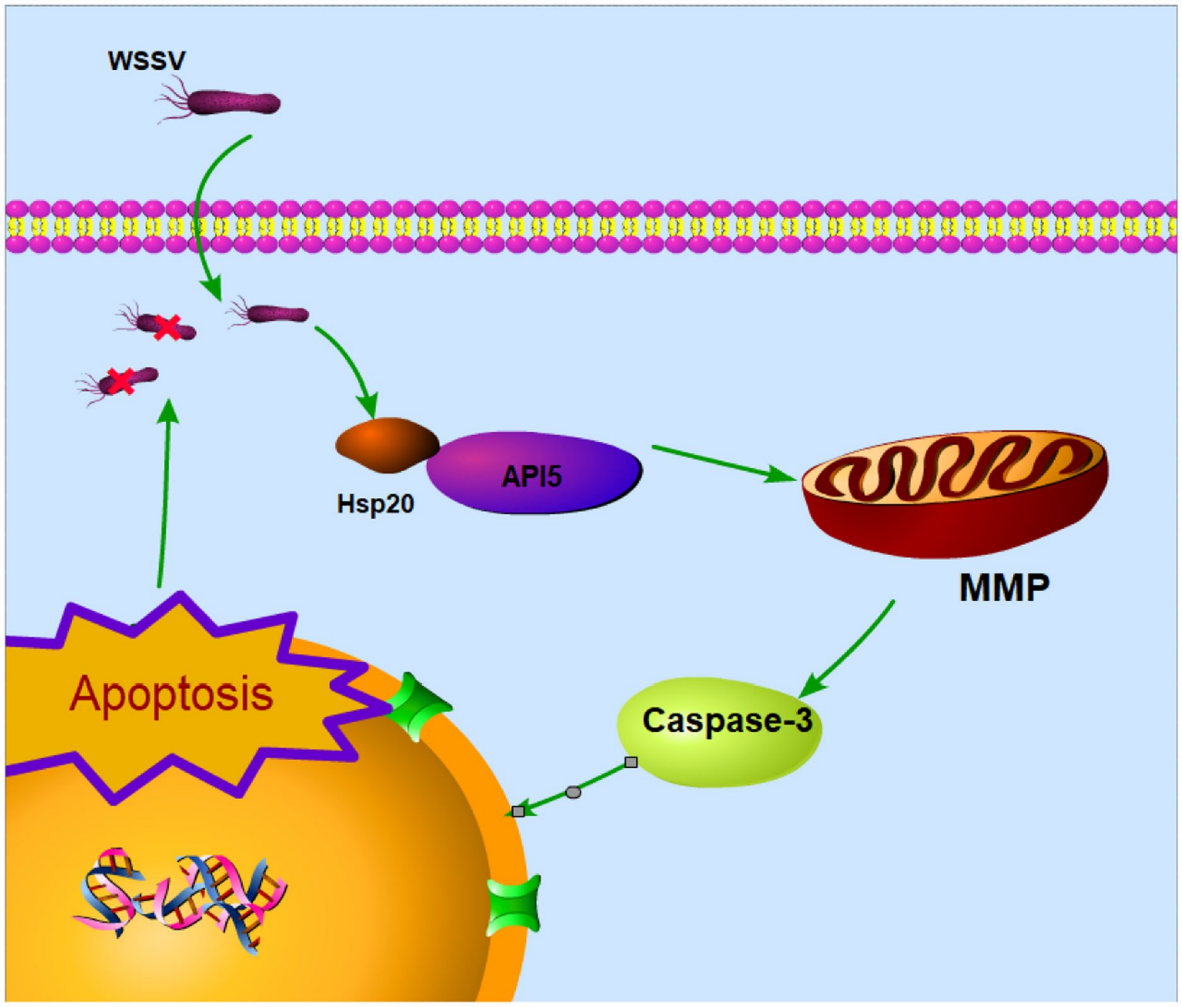
Proposed schematic diagram for the API5-Hsp20 axis-mediated apoptosis and virus infection regulation in mud crab.

In summary, these experiments provide further evidence that Hsp20 promotes apoptosis and reduces viral copy numbers by inhibiting the expression of API5. This also underscores the significant role of API5-Hsp20 axis in the antiviral process of mud crab (Figure 8).

## Materials and methods

### Mud crabs culture and WSSV challenge

Healthy mud crabs (about 45 g each) were sourced from a hatchery (Shantou, Guangdong, China) and acclimatized in the clean tanks for 3 days under laboratory conditions (temperature of 25°C and water salinity of 10‰). Before challenges, mud crabs were randomly checked to confirm absence of WSSV by using WSSV-specific primers and TaqMan probes (Table 1). In the infection experiment, mud crabs were injected with 200 μL of WSSV (10^3^ copies/μL) through the foot joint. At different times post-infection, the hemocytes and tissues of crabs were collected and stored for later use.

**Table 1:** Primers used in this study.

Primer name	Sequence (5′-3′)	Objective	Gene bank accession number
exAPI5-F	ccctgggatccccggaattcGAGCAAGAGATAGGAGAGACCA	Recombinant expression	OR670539
exAPI5-R	tcacgatgcggccgctcgagTCAGGTCAGGTCAGGTCAACAG		
PGEX-F	CCATCCTCCAAAATCGGATC	Recombinant expression	M21676.1
PGEX-R	GCCGCATCGTGACTGACTG		
βactin-F	CAGCCTTCCTTCCTGGGTATGG	qRT-PCR	XM_027353130
βactin-R	GAGGGAGCGAGGGCAGTGATT		
RT-API5-F	GCCTCACATCCCAGTTTATCGC	qRT-PCR	OR670539
RT-API5-R	CCACATCTTCATCCTCGCACA		
API5-Oligo-1	GATCACTAATACGACTCACTATAGGGCACCAATCCTCGATGTGCTCAAGATTT	RNAi	OR670539
API5-Oligo-2	AAATCTTGAGCACATCGAGGATTGGTGCCCTATAGTGAGTCGTATTAGTGATC		
API5-Oligo-3	AACACCAATCCTCGATGTGCTCAAGATCCCTATAGTGAGTCGTATTAGTGATC		
API5-Oligo-4	GATCACTAATACGACTCACTATAGGGATCTTGAGCACATCGAGGATTGGTGTT		
Q-WSSV-F	TTGGTTTCATGCCCGAGATT	qRT-PCR	MN840357.1
Q-WSSV-R	CCTTGGTCAGCCCCTTGA		
WSSV-probe	FAM-TGCTGCCGTCTCCAA-TAMRA	qRT-PCR	
GFP-Oligo-1	GATCACTAATACGACTCACTATAGGGGGCTACGTCCAGGAGCGCACCTT	RNAi	LN515608.1
GFP-Oligo-2	AAGGTGCGCTCCTGGACGTAGCCCCCTATAGTGAGTCGTATTAGTGATC		
GFP-Oligo-3	AAGGCTACGTCCAGGAGCGCACCCCCTATAGTGAGTCGTATTAGTGATC		
GFP-Oligo-4	GATCACTAATACGACTCACTATAGGGGGTGCGCTCCTGGACGTAGCCTT		
RT-Hsp20-F	ACCCCACCTTTGGACAG	qRT-PCR	FJ774659.1
RT-Hsp20-R	CCTCAGGCTTGGCACTC		
Hsp20-Oligo-1	GATCACTAATACGACTCACTATAGGGCAGTGAGGTCGTGATGGAGACAGAATT	RNAi	FJ774659.1
Hsp20-Oligo-2	AATTCTGTCTCCATCACGACCTCACTGCCCTATAGTGAGTCGTATTAGTGATC		
Hsp20-Oligo-3	AACAGTGAGGTCGTGATGGAGACAGAACCCTATAGTGAGTCGTATTAGTGATC		
Hsp20-Oligo-4	GATCACTAATACGACTCACTATAGGGTTCTGTCTCCATCACGACCTCACTGTT		

### Mud crab tissue harvest and hemocytes isolation

Firstly, the hemolymph of mud crabs was extracted in equal proportion to ACD anticoagulant, The hemocytes were mixed, 600 × g, centrifuged at 4 degrees for 10 min, and were used for total RNA extraction and protein sample preparation. Then the shell of the crab was opened to remove the inner lining, Gill, muscle, hepatopancreas, heart, intestine and other tissues were frozen in a centrifugal tube with liquid nitrogen and stored at −80°C for reserve. Then each tissue was ground in a mortar, and excess tissue fragments were filtered by a 70 μm cell screen to obtain cell homogenates for RNA and protein extraction.

### Biological information analysis of SpAPI5 and SpHsp20

The open reading frames (ORFs) of *Sp*API5 and *Sp*Hsp20 were initially predicted using the ORF Finder online tool.^1^ Then, the amino acid sequences within these ORFs were predicted using DNAMAN software. The functional domains of *Sp*API5 and *Sp*Hsp20 were determined by the utilization of Smart online tool.^2^ Besides, the tertiary structures of these proteins were predicted using AlphaFold 2 online tool.^3^ The evolutionary tree constructed by DNAMAN software was employed to analyze the diversity of *Sp*API5 across different organisms.

### The prokaryotic expression and protein purification of SpAPI5

The ORF of *Sp*API5 cDNA was amplified using PCR (the optimal temperature determined by temperature gradient PCR), employing specific primers designed based on the homologous sequence of pGEX-6P-1 plasmid and restriction enzyme EcoRI and XhoI. The PCR product with correct sequencing data was linked to pGEX-6P-1 plasmid and subsequently transfected into competent BL-21 *Escherichia coli* cells. After spreading the bacteria onto solid LB medium with ampicillin resistance, the resulting colonies were picked, expanded, and screened using primers to confirm successful plasmid integration. Positive clones were then selected for API5 protein expression. The *E. coli* containing the SpAPI5 recombinant plasmid was cultured at 37°C until reaching an OD600 of 0.6. Subsequently, IPTG (0.5 mM) was added and incubated with the cells at 12°C for 12 h. After centrifugation (8,000 × g, 10 min, 4°C), the collected cells were sonicated for 40 min using an ultrasonicator. Finally, the API5 protein was immobilized on GST resin and subsequently eluted from the resin using a reducing glutathione peptide solution (10 mM). The purity of the eluted API5 protein was assessed and the concentration was determined using SDS-PAGE.

### GST-tag pull-down and protein identification

GST-API5 was adsorbed on the resin of GST label, and the GST-API5-resin complex was incubated with the lysate of mud crab hemocytes with WSSV infected. The control group was GST-resin complex. After incubation, the bound proteins on the resin were eluted, SDS-Page electrophoresis was carried out, and then SDS-PAGE was stained with silver nitrate. Finally, after comparative analysis, different protein bands were collected and the protein types were identified by LC-ESI-MSMS (Thermo Scientific LTQ-Orbitrap Elite). Relevant parameters are as follows: Data dependent acquisition(DDA); Spray Voltage:2.0 kV; Capillary Temp:280°C; S-lens:60%; NCE:35% CID; Polarity:Positive; Resolution: Scan Event 1: 60,000@m/z 200, Scan Event 2: Ion trap; Max Injection Time: full MS 100 ms; Mass Range: m/z 350–1800; Full scan range; Top N: Top 20; Isolation window: 2.0 Da; Dynamic exclusion duration: 30 s.

### RNA interference assays

RNA interference (RNAi) was employed to the silence API5 in hemocytes. The siRNA primers were designed using BLOCK-iT RNAi Designer^4^ and synthesized using the T7 High Yield RNA Synthesis Kit (Takara, Japan). 50 μg of the specific siRNAs were injected into mud crab for 24 h, GFP-specific siRNA at the same dose served as a control, then, hemocytes were collected and subjected to qRT-PCR and Western blotting to evaluate the efficiency.

### Analysis of mRNA levels and WSSV copies

The qRT-PCR technique was employed to quantify the mRNA level and the copy numbers of WSSV in mud crab. The binding region of API5 primers was between 128 bp–229 bp, the binding region of Hsp20 primers was between 409 bp–492 bp, and the binding region of WSSV primers was between 231,144 bp–231200 bp. Briefly, the muscle tissue from WSSV-infected mud crab was collected, and genomic DNA was extracted using a genomic DNA extraction kit (TIANGEN, China). For qRT-PCR, TaqMan probes along with WSSV-specific primers were utilized. The formula of the standard curve: *y* = 1*10^(12.34624–0.292*Cp).

### FITC annexin V apoptosis detection

Cellular apoptosis rates of crab hemocytes were assessed through flow cytometry using FITC Annexin V Apoptosis Detection Kit (BD Pharmingen, United States). Initially, samples were subjected to centrifugation (600 × g, 10 min, 4°C), and then washed by washes PBS. Subsequently, a secondary centrifugation step (550 × g, 5 min, 4°C) was performed to recollect the hemocytes. The hemocytes were then dissolved in 100 μL of 1× binding buffer, and following the procedures outlined in the FITC Annexin V Apoptosis Detection Kit manual, 5 μL of PI and 5 μL of FITC Annexin V were sequentially added. After incubating these cells at room temperature in darkness for 15 min, cellular apoptosis rates were determined using flow cytometry. 10,000 particles were absorbed by flow cytometry. We selected particles with SSC value between 20,000 and 700,000 and FSC value between 200,000 and 2,400,000, and then analyzed the proportion of PE and FITC fluorescent particles.

### Caspase 3 activity detection

Caspase 3 activity was evaluated by Caspase 3 Activity Assay Kit (Beyotime, China) according to the manufacturer’s instructions. Initially, cells were subjected to centrifugation (600 × g, 10 min, 4°C), then, the cells were resuspended in 1 × PBS and centrifuged (600 × g, 5 min, 4°C) to remove the supernatant. 300 μL of lysis buffer was added to the cells and lysed for 15 min on ice. After that, the lysate was centrifugated (16,000 × g, 10 min, 4°C) to collect the supernatant. Finally, 65 μL of sample buffer, 25 μL of the sample, and 10 μL of Ac-DEVD-PNA (2 mM) were added to a 96-well ELISA plate and incubated at 37°C for 60 min, the absorbance of the samples at 405 nm was measured using a microplate reader.

### Mitochondrial membrane potential (Δψm) measurement

The membrane potential of hemocytes was measured by JC-1 Mitochondrial Membrane Potential Assay Kit (Beyotime, China) accordingly. Initially, hemocytes were collected via centrifugation (600 × g, 5 min, 4°C) and gently suspended in a 1 × PBS solution, followed by centrifugation (550 × g, 5 min, 4°C) to remove the supernatant. Afterward, 0.5 mL of JC-1 staining solution was added to the hemocytes and incubated at 37°C for 30 min. Subsequent to the incubation, the cells were washed twice with JC-1 staining buffer. Finally, fluorescence intensity at excitation wavelengths of 530 and 590 nm was measured using a Microplate Reader, and the same samples were observed using a confocal microscope (ZEISS, Germany), The FAM (Excitation wavelength: 460 nm; Emission wavelength: 515 nm) fluorescence channel was selected to observe the JC-1 monomer and the Alex 610 (Excitation wavelength: 488 nm; Emission wavelength: 628 nm) channel was used to observe the JC-1 aggregate.

### Immunoprecipitation and co-immunoprecipitation assay

*Sp*API5 antibody was purified using protein A/G beads methods. Next, beads were washed with PBS and resuspend them. The following is adding the purified antibody to the bead suspension and incubate at 4°C for 8 h with gentle shaking. When incubation is complete, antibody-coupled beads were washed with PBS to remove any unbound antibody. Then, the lysate and the target protein were added to the antibody-coupled beads and incubate at 25°C for 2 h with gentle shaking. After that, the beads were washed several times with a PBS containing 0.1% Tween-20 to remove nonspecifically bound proteins. The following was adding an SDS-PAGE sample buffer to the beads and incubate at 25°C for 1 h with gentle shaking. Then, the supernatant containing the immunoprecipitated protein was collected.

### Gray value analysis for western blot

The results of all the western blot experiments were analyzed with Image J gray values to represent the relative expression of proteins. First, import the resulting image into the Image J software, then set the image format to 8-bit, and click process to substract the background. Next, the strip color is reversed to white, and using the square image selection tool, the brightest strip in the result was selected and its relative brightness value is calculated. Then, a box of the same size is wrapped around each strip and its brightness value is calculated. Finally, the first strip is selected as a reference to calculate the relative brightness of the remaining strips.

### Statistical analysis

All experiments in our study were conducted in triplicate. The results are presented as means ± standard deviation (S.D.) and were analyzed using PRISM 6 software (GraphPad, San Diego, CA) as guided. The statistical analysis comprised the application of either the student’s *t*-test or one-way (or two-way) analysis of variance (ANOVA). A significance level of *p*-values <0.05 was considered statistically significant.

## Discussion

Apoptosis is a well-studied biological process which possess essential role for the maintenance tissue homeostasis through superfluous or potentially harmful cells in multicellular animals (Hengartner, 2000; Sung and Lee, 2005). API5 has been extensively studied in mammals, most of which are related to its apoptotic regulatory functions. For example, when humans are infected with influenza A virus, the viral protein can promote apoptosis by negatively regulating API5 (Mayank et al., 2015). The mechanism of API5 inhibiting apoptosis has also been studied recently. In mammals. API-5 was found to inhibit apoptosis of hepatoma cells through NF-κB pathway (Ren et al., 2010), and E2F1-dependent apoptotic pathway (Mayank et al., 2015). Furthermore, API5 initially recognized for its role in inhibiting cell apoptosis upon growth factor deprivation, emerges as a significant component of the immune defense against viral infections (Li et al., 2015). However, the role of API5 has not been addressed in invertebrate yet, especially in marine crustaceans. In this study, the results of multi-species comparison of amino acid sequences showed that API5 was conserved among species. Therefore, we infer that its function is also conservative. For the first time, our study found that API5 could inhibit apoptosis in mud crabs by weakening Caspase3 activity and decreasing mitochondrial membrane potential, thus promoting viral replication *in vivo*, which provides a new theoretical basis for understanding antiviral immune regulation in crustaceans.

Hsp20, a member of the small heat shock protein family, has garnered considerable attention in cancer research due to its roles in cell proliferation and apoptosis regulation (Karolczak-Bayatti et al., 2011). Apoptosis-related pathways involved in Hsp20 have also been reported, Hsp20 induces apoptosis through Fas/FasL (Zhong et al., 2015) and Akt (Li et al., 2019) pathway. So far, studies relevant to the mechanism of Hsp20-mediated apoptosis have been very scarce. In this study, we identified a hitherto unexplored interaction between API5 and Hsp20. Surprisingly, our research demonstrated that Hsp20 can suppress the expression of API5 through their mutual interaction, revealing a previously unknown regulatory mechanism. Besides, the antiviral immunological role of Hsp20 has not been addressed. While in our study, we found that Hsp20 could reduce viral copy numbers by inhibiting the expression of API5 during WSSV infection in mud crab. However, we observed simultaneous induction of API5 and Hsp20 expression in hemocytes after WSSV infection, which possess the completely opposite apoptotic function. Although API5 and Hsp20 showed the same variation trend after virus infection, the up-regulation amplitude of API5 was much larger than that of Hsp20 from both mRNA and protein levels, indicating that API5 and Hsp20 were mutually antagonistic, at the same time, 24 h after virus infection, the virus could inhibit apoptosis through the high expression of API5, thus exercising its immune escape function. The change of virus copy number after 0–96 h of challenge can also explain this problem, which is consistent with the infection process of WSSV in mud crabs. This groundbreaking discovery adds a new layer of complexity to our understanding of API5-Hsp20 axis, particularly in the context of apoptosis regulation during viral infections.

In summary, our study represents a pioneering exploration of API5- Hsp20 axis in mud crabs, shedding light on their roles in the immune response against viral infections. The revelation of API5 anti-apoptotic function and its modulation by Hsp20 provides a foundation for future research into the intricate mechanisms of immune regulation in marine invertebrates. This work underscores the importance of studying lesser-known species to uncover novel insights with potential implications for broader fields such as immunology and antiviral strategies. As we continue to delve into the complexities of immune responses in diverse organisms, further discoveries are likely to emerge, deepening our understanding of host-pathogen interactions and immune regulation across the animals.

## Data availability statement

The original contributions presented in the study are included in the article/supplementary material, further inquiries can be directed to the corresponding author.

## Ethics statement

The manuscript presents research on animals that do not require ethical approval for their study.

## Author contributions

HH: Data curation, Investigation, Writing – original draft. ND: Data curation, Investigation, Writing – original draft. XZ: Methodology, Writing – original draft. CY: Methodology, Writing – original draft. WW: Methodology, Writing – original draft. YG: Data curation, Funding acquisition, Investigation, Writing – original draft, Writing – review & editing.

## Footnotes

^1^ https://www.ncbi.nlm.nih.gov/orffinder/

^2^ http://smart.embl-heidelberg.de/

^3^https://colab.research.go ogle.com/github/sokrypton/ColabFold/blob/main/AlphaFold2

^4^ https://rnaidesigner.thermofisher.com/
